# Preliminary Safety Assessment for Mandarin Orange Peel Administration to Dogs Based on Physical Conditions and Blood Examination Parameters

**DOI:** 10.3390/metabo16030213

**Published:** 2026-03-23

**Authors:** Tomohiro Yonezawa, Yixue Lei, Cris Niño Bon B. Marasigan, Mao Komori, Nanasa Fujiwara, Jun Nakahigashi, Eiji Kobayashi

**Affiliations:** 1 Laboratory of Veterinary Clinical Pathology, Graduate School of Agricultural and Life Sciences, The University of Tokyo, 1-1-1, Yayoi, Bunkyo-ku, Tokyo 113-8657, Japan; 2 Wellness Development Research Center, AIR WATER INC., 1-7, Tsukisamu Higashi 2-jo 16-chome, Toyohira-ku, Sapporo 062-0052, Japan; nakahigashi-jun@awi.co.jp; 3 Kobayashi Regenerative Research Institute, LLC, 1 Chayano-cho, Wakayama-shi 640-8263, Japan

**Keywords:** mandarin orange peel (MOP), safety assessment, hesperidin, nobiletin, canine cognitive dysfunction syndrome, dose-escalation study, pesticide residues, psoralens

## Abstract

**Highlights:**

**What are the main findings?**

- Mandarin orange peel (MOP) administration at 2–10 g/head/day was well tolerated in healthy Beagle dogs.
- No significant dose-dependent abnormalities were observed in clinical signs, hematology, or biochemistry.

**What are the implications of the main findings?**

- MOP is a safe dietary intervention for older dogs, even at doses exceeding anticipated clinical levels.
- Comprehensive residue and composition screening confirms its suitability for use in canine cognitive support.

**Abstract:**

Background/Objectives: Mandarin orange peel (MOP) is rich in bioactive polymethoxyflavones, including hesperidin and nobiletin, which have shown neuroprotective effects in rodent models. However, comprehensive safety data in dogs are required to support its development as a therapeutic intervention for canine cognitive dysfunction syndrome. In this study, the safety profile of a standardized MOP formulation was evaluated in four healthy Beagle dogs. Methods: Initially, compositional analysis was performed, and 202 pesticide residues and psoralens were screened to ensure compliance with Japanese pet food safety standards. Subsequently, a dose-escalation study was conducted in which dogs received oral MOP at 2, 6, and 10 g/head/day for 3–4 weeks at each dose level. Clinical signs, hematology, and serum biochemistry were monitored throughout the study period. Results: The MOP powder composition and residue levels remained within regulatory safety limits. In the dose-escalation study, no significant dose-dependent abnormalities were observed in physical or clinicopathological parameters. One dog exhibited transient loose stools at higher doses and a temporary elevation in alkaline phosphatase levels at 2 g/head/day; however, these symptoms resolved spontaneously despite continued administration. Conclusions: MOP was safe and well tolerated in dogs even at 10 g/head/day (787–952 mg/kg/day), which is approximately five times the anticipated clinical dose. The observed fluctuations in active ingredient concentrations remained within the acceptable range for natural products and did not affect overall safety. Combined with comprehensive screening for residues, these results indicate that MOP is a high-quality and safe dietary intervention for older dogs.

## 1. Introduction

Mandarin orange peel (MOP), a traditional component of East Asian medicine, is rich in bioactive polymethoxyflavones and glycosides, particularly hesperidin and nobiletin [1,2,3,4]. Recent rodent studies have highlighted the neuroprotective [5], anti-inflammatory [6,7], and antioxidant properties of these compounds [2,3], suggesting their potential efficacy in treating age-related cognitive diseases [8]. The therapeutic benefits of MOP-derived flavonoids have been well-documented in laboratory rodents; nevertheless, their physiological effects and metabolic fate in companion animals, particularly dogs, remain insufficiently explored. Moreover, the variation in flavonoid composition among different MOPs remains incompletely characterized. Although rodent models have highlighted the neuroprotective potential of hesperidin and nobiletin, their findings cannot be directly extrapolated to canines. Significant interspecies differences in xenobiotic metabolism—particularly in glucuronidation and sensitivity to citrus-derived compounds, including limonene and psoralens [9,10]—necessitate independent safety assessments to establish appropriate dosing and mitigate potential systemic toxicity in dogs.

With the increasing life span of companion dogs, the prevalence of canine cognitive dysfunction syndrome and other age-associated metabolic disorders has increased [11]. Dietary interventions using natural antioxidants and prebiotics present a promising approach to enhancing the health of older dogs [12]. Hesperidin and nobiletin are hypothesized to act through direct systemic absorption and the modulation of the gut–brain axis via changes in the intestinal microbiota [13,14,15]. However, translating these benefits from rodents to canines requires rigorous safety assessments, as dogs exhibit unique metabolic pathways and sensitivities to certain plant-derived compounds such as limonene, which is frequently present in citrus extracts.

A recent pilot study showed that administering MOP orally to Beagle dogs resulted in significant shifts in the gut microbiota, including increased *Eggerthellaceae* and decreased *Fusobacteriaceae*, with preliminary signs of behavioral improvement without adverse gastrointestinal effects [8]. However, in that study, the safety endpoints were not systematically assessed because the focus was limited to microbiota and behavioral shifts. Comprehensive safety data regarding the prolonged MOP administration and its effect on systemic organ function in dogs are necessary to support its use as a commercial dietary supplement. Furthermore, verifying that MOP complies with the ingredient standards in the Japanese Act on Ensuring the Safety of Pet Food is a critical requirement for its future commercialization as a dietary product [16].

In this study, we conducted ingredient analysis and toxicological evaluation in accordance with the Act on Ensuring Pet Food Safety, along with a preliminary safety assessment for MOP administration using experimental dogs. The MOP used in this test was subjected to a safety-related compositional assessment, including analyzing the presence or absence of residual pesticides and other relevant components. Subsequently, a dose-escalation study was performed where dogs weighing approximately 10 kg received oral MOP at 2, 6, and 10 g/head/day for 3–4 weeks at each dose level. Clinical signs, hematology, and serum biochemistry were monitored throughout the study period. Notably, the test product was a dietary supplement for dogs, and the study was not conducted in accordance with clinical trial guidelines for pharmaceutical-grade products [17].

## 2. Materials and Methods

### 2.1. Preparation and Component Analysis of MOP

#### 2.1.1. Preparation of MOP Powder

Dried MOP chips (KISHU SHOKUHIN Co., Ltd., Wakayama, Japan) were used as samples and finely ground with a micropulverizer (ACM Pulverizer; HOSOKAWA MICRON CORPORATION, Osaka, Japan) to obtain approximately 50 kg of powder. The powder was subsequently sterilized at 90 °C for 120 min by a contracted processing company (Metal Color Co., Ltd., Osaka, Japan). MOP was prepared using an early maturing cultivar from Wakayama Prefecture, Japan, which was harvested in October 2024.

#### 2.1.2. Flavonoid Analysis (Hesperidin and Nobiletin)

Hesperidin (purity ≥ 98.5%) and nobiletin (purity ≥ 98%) standards were purchased from Funakoshi Co., Ltd., Tokyo, Japan. Liquid chromatography–mass spectroscopy (LC-MS) grade methanol (Kanto Chemical Co., Inc., Tokyo, Japan) was used in this study. Component analysis using high-performance liquid chromatography (HPLC) was performed to measure hesperidin and nobiletin in the powder.

A 200 mg portion of MOP powder was accurately weighed and extracted with 10 mL of methanol by refluxing at 80 °C for 1 h. The supernatant was transferred to a 20 mL volumetric flask. The residue was re-extracted with 10 mL of methanol under identical conditions. The combined extracts were adjusted to a final volume of 20 mL [17,18].

A 2 μL aliquot of this solution was filtered, diluted as necessary, and injected into an HPLC column (Agilent 1200 Series, Agilent Technologies, Inc., Santa Clara, CA, USA). The analytical column employed was a Fusion C30 column (2.0 × 100 mm, 3 μm; Nomura Chemical Co., Ltd., Aichi, Japan). The mobile phase comprised 10 mM aqueous phosphoric acid and methanol at a flow rate of 0.5 mL/min. The gradient program for hesperidin was as follows: 20% methanol at 0–1 min, 20–40% at 1–4 min, 40–60% at 4–15 min, and 60–95% at 15–16 min, followed by a 4 min hold at 95% methanol. For nobiletin, the gradient was initiated at 50% methanol, increased from 50% to 95% over 0–15 min, and was maintained for 5 min. The column temperature was maintained at 40 °C, and detection was conducted at 285 nm. A methanol solution prepared in a similar manner but without the sample served as the blank.

#### 2.1.3. Component Analyses (Residual Pesticides, the Japanese Act on Ensuring the Safety of Pet Food, Guaranteed Components, and Psoralen)

The residual pesticide analysis was outsourced to Eurofins QKEN Ltd. (Fukuoka, Japan). In compliance with the Japanese Act on Ensuring the Safety of Pet Food, analyses were conducted by Japan Food Research Laboratories (JFRL, Tokyo, Japan). Reportedly, psoralen, which is primarily present in citrus fruits, such as lemons, causes adverse symptoms, including vomiting and diarrhea in dogs (American Society for the Prevention of Cruelty to Animals, ASPCA). Therefore, psoralen was quantified by the JFRL. A 1 g sample was extracted with 100 mL of methanol by refluxing at 80 °C for 1 h. The supernatant was transferred to a 250 mL volumetric flask. The residue was re-extracted with methanol (100 mL) under the same conditions, and the combined extracts were adjusted to a final volume of 250 mL. A 1 mL aliquot of this solution was diluted to 10 mL and analyzed using LC-MS. Chromatographic separation was achieved using an Inertsil ODS-2 column (2.1 × 150 mm, 5 μm; GL Sciences Inc., Tokyo, Japan). The mobile phase comprised a mixture of 1% aqueous acetic acid and acetonitrile (60:40) delivered at a flow rate of 0.2 mL/min. The column temperature was maintained at 30 °C. Detection was performed through electrospray ionization in the positive mode, monitoring the *m*/*z* transition from 187.2 to 115.0.

### 2.2. Safety Assessment of MOP in Clinically Healthy Dogs

#### 2.2.1. Animals

All experimental procedures were performed following the guidelines of the Regulations of Experimental Animal Administration in Japan and approved by the Animal Care and Use Committee of the Graduate School of Agricultural and Life Sciences, University of Tokyo (P25-008). Four clinically healthy beagles (*n* = 4) were included. They were all aged 1–2 years, with two intact males (#1 and #2) and two intact females (#3 and #4). Their average weight at the start of the study was 10.8 kg (range 10.4–11.5 kg), increasing to 12.0 kg (range 11.1–12.7 kg) by the end. The animals were housed in a temperature-controlled room (23 ± 3 °C) under a 12-h light/dark cycle. All dogs had *ad libitum* access to tap water and were fed a normal commercial diet (DS-A; Oriental Yeast, Tokyo, Japan). General clinical examinations were performed before the study to confirm the absence of comorbidities.

#### 2.2.2. Experimental Designs

**Figure 1:**
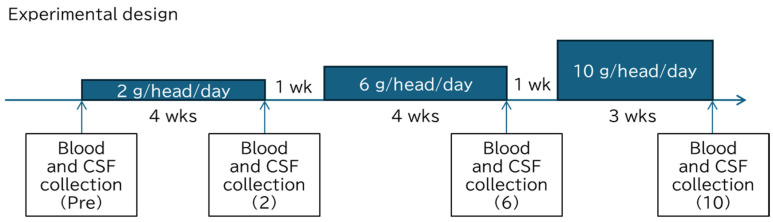
Experimental design for the safety assessment of MOP in clinically healthy dogs. The black areas indicate the administration period. The intervening 1-week denotes the washout period. The letters in parentheses for each sample collection correspond to the labels on the horizontal axes in Figure 2 and Figure 3. CSF, cerebrospinal fluid. The dogs weighed 10.3–12.5 kg. The actual calculated doses are described in Section 3.2.

A dose escalation study was conducted using MOP formulated as capsules, gelatin cubes, or suspensions. The MOP provided was a gelatin block containing 2 g of MOP. The doses were planned by working backward from the doses actually intended for clinical use in dogs. All four dogs weighed approximately 10 kg, and each was scheduled to orally receive 1, 3, or 5 blocks per day (2, 6, or 10 g/head/day) (Figure 1). MOP was administered twice daily in divided doses with dry food. When feeding 1 g/head one time, half of a block was given. The actual calculated doses are described in the Results. The doses were escalated in an orderly manner after a 1-week washout period. Clinical signs, including activity level, appetite, vomiting, fecal consistency, water intake, and urine color, were monitored daily. A comprehensive physical examination was performed weekly to record the body weight, heart rate, rectal temperature, and coat condition. Blood and cerebrospinal fluid (CSF) samples were collected at the end of each stage.

**Figure 2:**
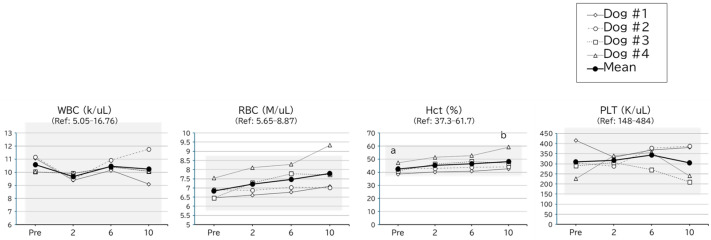
CBC results for the safety assessment of MOP in clinically healthy dogs. Pre, 2, 6, and 10 represent the time before initiating administration and completing dosing at 2, 6, and 10 g/head/day, respectively. The white diamonds, circles, squares, and triangles correspond to dogs #1, #2, #3, and #4, respectively. The thick black line and black circles indicate the average. Ref indicates the reference range for the measurement item. RBC, red blood cell number; Hct, hematocrit; WBC, white blood cell number; PLT, platelet number; CBC, complete blood count. Letters indicate significant differences determined by Dunn’s test following Friedman’s test (*p* < 0.05).

**Figure 3:**
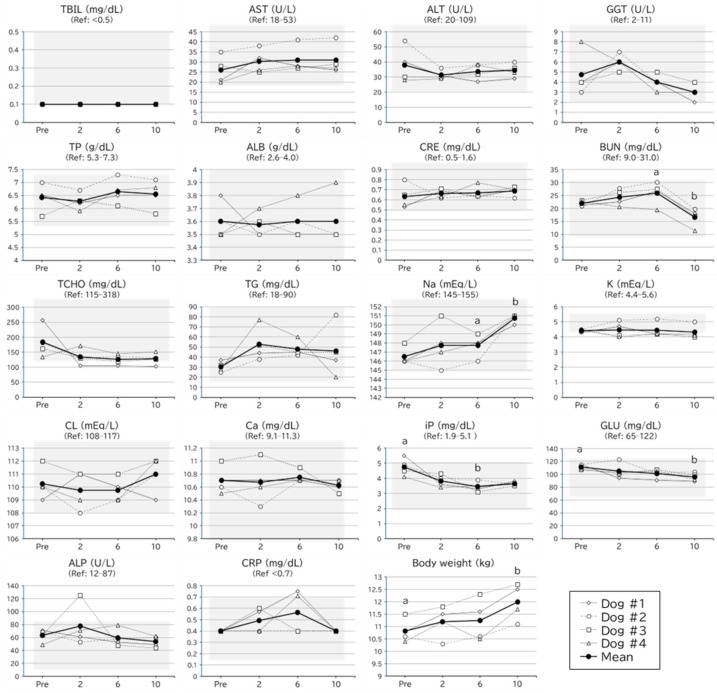
Blood chemistry examination results and body weight for the safety assessment of MOP in clinically healthy dogs. Pre, 2, 6, and 10 represent the time before initiating administration and completing dosing at 2, 6, and 10 g/head/day, respectively. The white diamonds, circles, squares, and triangles correspond to dogs #1, #2, #3, and #4, respectively. The thick black line and black circles indicate the average. Ref indicates the reference range for the measurement item. TBIL, total bilirubin; AST, aspartate aminotransferase; ALT, alanine aminotransferase; GGT, gamma glutamyl transferase; TP, total protein; ALB, albumin; CRE, creatinine; BUN, blood urea nitrogen; TCHO, total cholesterol; TG, triglycerides; Na, sodium; K, potassium; CL, chloride; Ca, calcium; iP, inorganic phosphate; GLU, glucose; ALP, alkaline phosphatase; CRP, C-reactive protein. Letters indicate significant differences determined by Dunn’s test following Friedman’s test (*p* < 0.05).

#### 2.2.3. Sample Collection

Blood samples were collected at the beginning and completion of each dosing period, with a maximum of 5 mL obtained per session from the jugular vein. CSF samples were collected while the patient was sedated, 3 h after the final administration of each dose. The dogs were sedated via intramuscular injection of a mixture of medetomidine hydrochloride (0.01 mg/kg) and midazolam (0.1 mg/kg). In the sedative state, vital signs, including heart rate and mucosal color, were closely monitored to ensure clinical safety. Upon completion of the procedure, sedation was immediately reversed using atipamezole hydrochloride injection (0.05 mg/kg) to facilitate a smooth and rapid recovery. Approximately 1 mL of CSF was harvested from the cisterna magna using standard techniques. Immediately after collection, blood samples were treated with anticoagulants (ethylenediaminetetraacetic acid and heparin) and analyzed for routine hematological and biochemical parameters using standard in-house laboratory protocols. CSF samples were promptly aliquoted and stored at −80 °C until further analysis.

#### 2.2.4. Hematological Examinations and CSF Metabolite Analysis

Complete blood count and blood chemistry parameters including levels of total protein, albumin, alanine aminotransferase, aspartate aminotransferase, alkaline phosphatase (ALP), gamma glutamyl transferase, total bilirubin, total cholesterol, triglyceride, ammonium, lipase, c-reactive protein, blood urea nitrogen (BUN), creatinine, calcium, inorganic phosphate, sodium (Na), potassium, and chloride were measured using automated analyzers (Procyte DX, IDEXX Laboratories, ME, USA; Dimension EXL 200, Siemens Healthineers, Munich, Germany). Supernatants of the CSF samples were analyzed using LC-MS to measure MOP metabolites.

For CSF metabolite analysis, standard compounds of hesperidin (purity ≥ 98.5%), homoeriodictyol (purity ≥ 98.0%), nobiletin (purity ≥ 98%), and an internal standard (IS) compound of apigenin-d5 (purity ≥ 99%) were purchased from Funakoshi Co., Ltd. (Tokyo, Japan). Standard compound of rac-hesperetin 7-O-β-D-glucuronide (purity ≥ 98%) was obtained from Cosmo Bio Co., Ltd. (Tokyo, Japan). Hesperetin (98.6% purity) was purchased from FUJIFILM Wako Pure Chemical Corporation (Osaka, Japan). Methanol (LC-MS grade) was obtained from Kanto Chemical Co., Inc. (Tokyo, Japan).

CSF samples (50 μL) were mixed with 150 μL of methanol containing the IS (37.5 ng), vortexed, and centrifuged at 12,000 rpm for 10 min. The resulting supernatant (5 μL) was injected into an Agilent 1260 Infinity II HPLC system coupled with an Agilent 6470 triple-quadrupole LC-MS (Agilent Technologies, Santa Clara, CA, USA). A Unison UK-C18 UP column (3 μm, 100 × 2 mm; Imtakt Corporation, Kyoto, Japan) was used for chromatographic separation. The mobile phases comprised 0.1% formic acid in water (A) and methanol (B). The flow rate was set to 0.4 mL/min. The gradient program was as follows: 10% B for 1.5 min, 10–100% B from 1.5 to 20 min, followed by an isocratic hold at 100% B for 5 min. The injection volume was 5 μL, and the column temperature was maintained at 40 °C. The mass spectrometer was operated in the multiple reaction monitoring mode. The monitored transitions were as follows: hesperidin (*m*/*z* 611.2–303.1), rac-hesperetin 7-O-β-D-glucuronide (*m*/*z* 479.1–303.1), homoeriodictyol (*m*/*z* 313.1–153.0), hesperetin (*m*/*z* 303.1–153.0), nobiletin (*m*/*z* 403.1–373.1), and apigenin-d5 (IS, *m*/*z* 276.1–156.0). A methanol solution processed in the same manner but without the sample served as the blank.

### 2.3. Statistical Analysis

Analyses were performed using the GraphPad Prism 10 software (GraphPad Software, Boston, MA, USA). Clinical and laboratory data were compared before and after drug administration using the Wilcoxon matched-pairs signed-rank test. For time-course evaluations, data were analyzed using the Friedman test, followed by Dunn’s test for multiple comparisons. Statistical significance was set at *p* < 0.05.

## 3. Results

### 3.1. Chemical Composition of MOP

Hesperidin and nobiletin were measured using HPLC to analyze the components of the MOP powder (Figures S1 and S2). The hesperidin and nobiletin contents of the MOP used in this study were 8.5 g/100 g and 47 mg/100 g, respectively. Additionally, although an external laboratory performed the analysis, the non-sterilized MOP was also examined and found to contain 8.4 g/100 g and 46 mg/100 g of hesperidin and nobiletin, respectively, indicating that the sterilization process did not reduce these components.

The HPLC method was validated for the quantification of both hesperidin and nobiletin. Linearity was confirmed using five calibration levels (1–20 μg/mL) for each analyte, with correlation coefficients exceeding 0.999 across the tested concentration ranges. The limits of detection (LOD) and quantification were determined based on signal-to-noise criteria and were 0.1 μg/mL and 1 μg/mL, respectively. Repeatability was evaluated using six replicate injections of a mid-level standard solution (10 μg/mL). The relative standard deviation values were 0.43% (*n* = 6) and 0.36% (*n* = 6) for hesperidin and nobiletin, respectively, demonstrating adequate precision of the analytical method.

Residual pesticides were analyzed by Eurofins QKEN Ltd., which conducts the largest number of pesticide residue tests in Japan and provides citrus-specific multi-residue analytical panels (Table 1). Of the 129 pesticides permitted for use in domestic citrus cultivation and available for analysis, 106 (82%) were included. Furthermore, 94 widely used, domestically registered pesticides and agents commonly utilized in agricultural practices, including dithianon and dithiocarbamates, were added. Overall, 202 chemical analytes were investigated. All detected values were below the regulatory limits (Table S1). The standards used in this study were those for dried citrus peel intended for human consumption, which is categorized as “other spices” under the Japanese Food Sanitation Act. All other parameters were below 0.01 mg/kg, and dithianon was present at concentrations < 0.1 mg/kg.

It was required that the analysis comply with the Japanese Act on Ensuring the Safety of Pet Food, including guaranteed proximate composition analysis and psoralen determination. Hence, they were conducted by JFRL, an independent third-party testing organization. All values of the ingredient compounds were within the acceptable limits of the Act (Table 2). The guaranteed proximate compositions of the MOP are also presented in Table 3. The psoralen content was below 0.5 mg/100 g (Figure S3).

### 3.2. Safety Assessment of MOP in Clinically Healthy Dogs

To evaluate the safety of MOP, gelatin blocks containing MOP were repeatedly administered to four clinically healthy dogs at doses of 2, 6, and 10 g/head/day for 3–4 weeks (Figure 1). These doses were equivalent to an average of 182 (range 169–194), 536 (488–583), and 864 (787–952) mg/kg/day, respectively. One of the four dogs (dog #1) preferred the MOP. One of the remaining dogs (dog #3) for the first 3 days and the other two (dog #2 and 4) for the first 10 days left around 30% of the MOP cubes at a dose of 2 g/head/day. However, when gelatin blocks containing MOP were thoroughly mixed with dry food, the dogs consumed the entire portion. None of the dogs exhibited any abnormalities in activity, appetite, or urination throughout the observation period. All four dogs had good stools (2 g/head/day). However, after 3 days of 6 g/head/day dose administration, one of the four dogs (dog #3) intermittently had loose stools for 11 of the 28 days and for 17 of the 21 days after initiating the 10 g/head/day dose. The loose stools were types 5 to mild 6 on the Bristol Stool Form Scale, ranging from soft blobs with clear-cut edges to fluffy pieces with ragged edges. Mushy (Type 6) or watery stools without solid pieces, classified as entirely liquid (Type 7), were not observed. No clinically significant weight loss was associated with the symptoms.

The results of the hematological examinations are shown in Figure 2 and Figure 3. Although statistical fluctuations were observed between the periods for several parameters, including hematocrit (Hct), Na, BUN, inorganic phosphorus (IP), and glucose (GLU), no clear dose-responsive changes were observed, and all variations were sporadic. Except for a single ALP value, all measurements remained within the reference range.

In Dog #3, an increase in ALP was transiently observed 1 month after administering a 2 g/head/day dose (Figure 3). This increase was subsequently resolved, with ALP levels returning to the reference range by the end of the 6 g/head/day dose administration. ALP levels remained within the unremarkable range throughout the 10 g/head/day dose administration period.

CSF target analytes were below the assay LODs. CSF collected 3 h after the final dose showed hesperidin concentrations of <10 ng/mL, while nobiletin, hesperetin, rac-hesperetin-7-O-β-D-glucuronide, and homoeriodictyol were all detected at concentrations < 2 ng/mL in all samples. These values indicate that all analytes were below the detection limits of the CSF assay. The relatively higher LODs observed in CSF were attributable to matrix-related interference from endogenous co-eluting components, which reduced signal-to-noise ratios and consequently elevated the effective detection thresholds. Although no analytes were quantifiable at the sampled time point, the results do not preclude the presence of central nervous system concentrations below the LOD or transient exposures occurring outside the sampling window.

## 4. Discussion

In our previous studies, MOP, administered as a dietary supplement for dogs, was shown to offer potential health benefits, particularly regarding intestinal health and cognitive function [8]. In the present study, before large-scale clinical trials, the safety of MOP was evaluated by quantifying its active ingredients and screening for drug residues and contaminants to ensure compliance with pet food safety standards. Repeated-dose studies using clinically healthy dogs confirmed the absence of any physical or clinicopathological abnormalities. Since hesperidin and nobiletin are considered the primary active components of MOP [8], we further verified whether the MOP used in this pet food formulation consistently contained sufficient concentrations of these compounds.

Recent pharmacological studies using small animal models have shown that hesperidin and nobiletin—bioactive compounds found in certain fruits—are effective against Alzheimer’s-like pathologies [19,20]. Reportedly, in mouse models, hesperidin reduces the elevated levels of phosphorylated tau (p-tau) protein [19], whereas nobiletin significantly mitigates beta-amyloid accumulation in the hippocampus and cortex, suggesting its potential for cognitive enhancement [18]. Furthermore, the existing literature suggests that mandarin peel containing these compounds helps alleviate dementia symptoms [5,21,22]. The clinical improvements observed in older dogs in our previous study were likely caused by consuming MOP, which is rich in these two flavonoids that appear to act through distinct pathways [8]. In the present study, the concentrations of hesperidin and nobiletin in the MOP extract were 8.5 g/100 g and 47 mg/100 g, respectively. Compared with the values reported in our previous study (9.3 g/100 g hesperidin and 41 mg/100 g nobiletin), the results showed 9% decrease and 15% increase in hesperidin and nobiletin, respectively. Such variations are typical of products derived from natural ingredients, where nutrient profiles fluctuate with harvest year and production lot. According to the Act on Ensuring the Safety of Pet Animals Feed [23], the concentration should be between 80% and 120% of the labeled value. Hence, we believe the variability in this case is entirely acceptable. These minor deviations were within the acceptable range for natural food-based ingredients.

Under the Food Labeling Act [23,24], it is mandated that guaranteed amounts are indicated on the product label. Stringent residue-concentration standards have been established for conventional foods; however, such regulations do not necessarily apply to supplements. Regulatory laxity is usually cited as a contributing factor in the frequent occurrence of supplement-related adverse events [25]. For MOP, which is primarily derived from discarded mandarin peels [8,26,27,28], particular attention should be paid to the potential presence of pesticide residues. Accordingly, 202 pesticide residues and other harmful substances were screened in this study. Pesticide evaluation was based on the individual regulatory limits; cumulative exposure from multiple low-level residues was not assessed, although the total burden was considered negligible given the trace levels detected. All results were below regulatory limits, indicating that MOP poses minimal risk in this regard. Furthermore, the excessive intake of psoralens, which are abundant in citrus fruits, is toxic to dogs [29]. The psoralen concentrations measured in this study were below 0.5 mg/100 g. Collectively, these findings suggest that dogs can safely consume MOP as a dietary supplement without any significant toxicological risk. Although our compositional analysis confirms compliance with regulatory safety limits established by the Japanese Act on Ensuring the Safety of Pet Food, it is important to note that these thresholds are primarily designed to mitigate the risks of acute toxicity. The potential for cumulative effects resulting from chronic, low-dose exposure to residual compounds remains a concern in the long-term administration of dietary supplements. The findings of this study represent an initial assessment, and further long-term longitudinal studies are warranted to fully characterize the impact of prolonged, low-dose ingestion.

A repeated-dose study was conducted in clinically healthy dogs to evaluate the safety and tolerability of MOP before formal clinical trials. They were administered MOP at a previously established effective dose of 2 g/head/day [8], and at exaggerated doses of 6 g/head/day and 10 g/head/day, with each dosing period lasting 3–4 weeks. At higher doses (6 and 10 g/head/day), mild and intermittent loose stools were observed in one of the four dogs. Notably, this condition was transient and did not persist throughout the study period. Previous studies have shown that MOP consumption can modulate intestinal microbiota, specifically characterized by decreased *Fusobacteriaceae* and increased *Eggerthellaceae* [8]. Such shifts in microbial distribution could temporarily alter stool consistency in some individuals, depending on their baseline gastrointestinal status. However, even at a maximum dose of 10 g/head/day, no severe clinical signs, including weight loss, hematochezia, or tenesmus, were identified. Regarding hepatic biomarkers, the same individual showed a transient increase in ALP levels during the 2 g/head/day administration phase. ALP levels spontaneously returned to baseline during the subsequent 6 and 10 g/head/day dosing periods. Given the lack of dose–response and the subsequent resolution despite increased exposure, a direct causal relationship between MOP administration and ALP elevation remains inconclusive. Based on these results, MOP is unlikely to pose a significant health risk to healthy dogs even at five times the anticipated clinical dose. Nevertheless, clinicians should exercise caution regarding potential gastrointestinal sensitivity and ALP fluctuations, particularly in individuals with preexisting chronic diarrhea and/or transient hepatic enzyme induction. Additionally, while statistical fluctuations were observed between the periods for several parameters, including Hct, Na, BUN, IP, and GLU, no clear dose-responsive changes were noted, and all variations were sporadic and within/peri reference range. As these observed fluctuations did not follow a clear, dose-dependent pattern, they are unlikely to indicate systemic toxicity; nevertheless, continued clinical monitoring remains prudent. As this safety assessment was not conducted following Good Clinical Practice guidelines in compliance with the Act on Securing Quality, Efficacy, and Safety of Products Including Pharmaceuticals and Medical Devices [30], the findings should be interpreted as preliminary and require further validation in controlled clinical settings.

Hesperidin, a prominent plant-derived flavanone, has been extensively studied for its neuroprotective effects in various neurodegenerative models, including Alzheimer’s disease [31], Parkinson’s disease [32], Huntington’s disease [33], and stroke-induced brain injury [34,35]. These beneficial properties are primarily attributed to its antioxidant, anti-inflammatory, and antiapoptotic activities. Given its lipophilic nature, reports have shown that it readily crosses the blood–brain barrier (BBB) [36]. In this study, we quantified hesperidin and its metabolites in canine CSF to provide definitive evidence of cerebral translocation. Previous literature has indicated that orally administering 4 g MOP to dogs yields plasma hesperidin concentrations of approximately 20 ng/mL. As hesperidin undergoes rapid metabolism to form glucuronide conjugates such as hesperetin and homoeriodictyol glucuronides [37], the parent compound and its major metabolites were targeted in our analysis. Unfortunately, neither hesperidin nor its metabolites were detected in canine CSF samples using the current LC-MS analytical framework. This lack of detection precludes definitive conclusions regarding the direct transport of these compounds across the BBB in dogs. Furthermore, hesperidin or its metabolites may not be present in high concentrations if they cross the BBB. Alternatively, the neuroprotective effects of MOP may be mediated through indirect pathways. It has also been hypothesized that modifications to the intestinal environment influence cerebral function and alleviate neurodysfunction [8]. The non-detection of hesperidin, nobiletin, and key metabolites in CSF at 3 h post-dose should be interpreted in the context of the analytical conditions. Citrus flavonoids undergo rapid intestinal and hepatic metabolism with extensive conjugation. Additionally, plasma protein binding and efflux transport at the BBB can reduce the unbound, brain-available fraction to levels below the LOD of our CSF assay. Therefore, these data neither confirm nor exclude direct central nervous system exposure at low concentrations. Moreover, while hesperidin has been reported to cross the BBB in rodent models, definitive canine-specific data regarding its BBB permeability remain unavailable.

It is also plausible—and consistent with our previous pilot study in dogs, which showed MOP-associated shifts in gut microbiota and behavioral tendencies—that the neurological benefits may arise predominantly via the gut–brain axis. This pathway may involve microbial biotransformation of flavonoids, short-chain fatty acid signaling, immune-neuroendocrine modulation, and vagal mechanisms. Further studies are required to clarify the mechanisms underlying the clinical benefits of MOP.

Building on these foundational safety data, clinical trials are underway, following formal approval from the Institutional Ethics Committee. To date, no adverse events have been identified, and preliminary observations on mitigating the symptoms of canine cognitive dysfunction syndrome are consistent with previously reported findings [8]. We anticipate that a comprehensive analysis of these clinical data will be summarized and reported in a forthcoming publication. Future research should aim to integrate untargeted metabolomics with longitudinal microbiome analysis to further elucidate the indirect gut–brain interactions hypothesized to underlie the clinical benefits of MOP.

This study had some limitations that should be considered when interpreting the results. The sample size was small (*n* = 4), and the investigation lacked a conventional untreated control group. While this design is common in pilot clinical evaluations of dietary supplements, it limits the statistical power and the ability to definitively generalize the safety findings to a broader population. The dosing duration of 3–4 weeks per level is shorter than the 90-day exposure period required by international regulatory guidelines, including OECD Test Guideline 409 for repeated-dose toxicity testing in non-rodents. Therefore, these findings should be considered preliminary clinical observations of tolerability rather than a standardized, comprehensive toxicological assessment. As this study was conducted in a veterinary clinical setting using food-grade ingredients, rather than in a strictly controlled laboratory environment, potential confounding factors, including individual metabolic variations or dietary influences, cannot be entirely excluded. Repeated sedation may affect physiological parameters. Despite these limitations, the absence of observable adverse effects provides initial evidence supporting the clinical safety of MOP as a dietary adjunct in dogs. The lack of a control group precludes a definitive causal attribution of all observed changes to MOP; therefore, these findings should be interpreted as a preliminary safety profile. Further large-scale, placebo-controlled studies with extended observation periods are warranted to confirm these preliminary findings.

## 5. Conclusions

This study showed that MOP is a safe dietary supplement for dogs, with no significant toxicological risk or persistent adverse effects, even at five times the anticipated clinical dose. Further investigations are required to clarify the precise clinical effects and fundamental mechanisms underlying MOP’s neuroprotective efficacy.

## Figures and Tables

**Table 1:** Residual pesticide in MOP.

Detected Component	Result	Regulatory Limit
	(mg/kg)	(mg/kg)
Tebuconazole	0.19	15
Fenpropathrin	0.04	10
Chlorfenapyr	0.03	10
Phenthoate	0.02	10
Buprofezin	0.01	10
Pyflubumide	0.11	5
Fluxametamide	0.05	4
Carbendazim/Benomyl/ Thiophanate-methyl (total)	0.06	3
Ethychlozate	0.01	15
Isoprothiolane	0.02	7
Spiromesifen	0.13	10
Clothianidin	0.03	10
Trifloxystrobin	0.04	10
Imidacloprid	0.01	5
Dinotefuran	0.12	10
Tolfenpyrad	0.01	15
Ethiprole	0.11	3
Dithiocarbamates *	2.8	10

MOP, Mandarin orange peel. * dithiocarbamates expressed as CS2, including maneb, mancozeb, metiram, propineb, thiram, and ziram.

**Table 2:** Regulated components of MOP under the Japanese Act on Ensuring the Safety of Pet Food.

Category	Item	Unit	Result	Standard Value
Additives	Sodium nitrite	g/t	<2	100
	Ethoxyquin	g/t	<1	Ethoxyquin: 75 Total: 150
	Dibutylhydroxytoluene	g/t	<1	--
	Butylhydroxyanisole	g/t	<1	--
Pesticide	Glyphosate	μg/g	<0.1	15
residues	Chlorpyrifos-methyl	μg/g	<0.1	10
	Pirimiphos-methyl	μg/g	<0.1	2
	Malathion	μg/g	<0.1	10
	Methamidophos	μg/g	<0.01	0.2
Contaminants	Aflatoxin B1	μg/g	<0.001	0.02
	Deoxynivalenol	μg/g	<0.05	2
	Cadmium	μg/g	<0.01	1
	Lead	μg/g	<0.05	3
	Arsenic	μg/g	<0.1	15
	BHC (sum of α-, β-, γ-, and δ-isomers)	μg/g	<0.002	0.01
	DDT (including DDD and DDE)	μg/g	<0.02	0.1
	Aldrin and dieldrin (sum)	μg/g	<0.002	0.01
	Endrin	μg/g	<0.002	0.01
	Heptachlor and heptachlor epoxide	μg/g	<0.002	0.01
Other	Melamine	μg/g	<0.5	2.5

MOP, Mandarin orange peel; BHC, benzene hexachloride (hexachlorocyclohexane, HCH); DDT, dichlorodiphenyltrichloroethane; DDD, dichlorodiphenyldichloroethane; DDE, dichlorodiphenyldichloroethylene.

**Table 3:** Guaranteed proximate compositions of MOP.

Item	Result (%)
Moisture	8.9
Crude protein	6.8
Crude fat	1.9
Crude fiber	1.3
Ash	3.7

MOP, Mandarin orange peel.

## Data Availability

The data presented in this study are available on request from the corresponding author due to the inclusion of analytical data from external institutions.

## Supplementary Materials

The following supporting information can be downloaded at: https://www.mdpi.com/article/10.3390/metabo16030213/s1, Table S1: List of Compounds for Residual Pesticide Analysis (202 Compounds); Figure S1: HPLC Chromatogram of Hesperidin, Figure S2: HPLC Chromatogram of Nobiletin, Figure S3: LC/MS Chromatogram of Psoralen, Figure S4: LC/MS Chromatogram from CSF Analysis.

## Abbreviations

The following abbreviations are used in this manuscript:

MOP	Mandarin Orange Peel
HPLC	High-Performance Liquid Chromatography
LC-MS	Liquid Chromatography–Mass Spectrometry
CSF	Cerebrospinal Fluid
IS	Internal Standard
BBB	Blood–Brain Barrier
ALP	Alkaline Phosphatase
JFRL	Japan Food Research Laboratories
Hct	Hematocrit
Na	Sodium
BUN	Blood Urea Nitrogen
IP	Inorganic Phosphorus
GLU	Glucose
LOD	Limits of Detection

## References

[1] Pyrzynska K. (2022). Hesperidin: A review on extraction methods, stability and biological activities. Nutrients.

[2] Chukwuma C.I. (2024). Antioxidative, metabolic and vascular medicinal potentials of natural products in the non-edible wastes of fruits belonging to the *Citrus* and *Prunus* Genera: A review. Plants.

[3] Escobedo-Avellaneda Z., Gutiérrez-Uribe J., Valdez-Fragoso A., Antonio Torres J., Welti-Chanes J. (2014). Phytochemicals and antioxidant activity of juice, flavedo, albedo and comminuted orange. J. Funct. Foods.

[4] Kubo M., Fujita T., Nishimura S., Tokunaga M., Matsuda H., Gato T., Tomohiro N., Sasaki K., Utsunomiya N. (2004). Seasonal variation in anti-allergic activity of citrus fruits and flavanone glycoside content. Nat. Med..

[5] Hajialyani M., Hosein Farzaei M., Echeverría J., Nabavi S.M., Uriarte E., Sobarzo-Sánchez E. (2019). Hesperidin as a neuroprotective agent: A review of animal and clinical evidence. Molecules.

[6] Al-Khayri J.M., Sahana G.R., Nagella P., Joseph B.V., Alessa F.M., Al-Mssallem M.Q. (2022). Flavonoids as potential anti-inflammatory molecules: A review. Molecules.

[7] Maleki S.J., Crespo J.F., Cabanillas B. (2019). Anti-inflammatory effects of flavonoids. Food Chem..

[8] Nakahigashi J., Kurikami M., Iwai S., Iwamoto S., Kobayashi S., Kobayashi E. (2025). Exploring the pharmacokinetics and gut microbiota modulation of hesperidin and nobiletin from mandarin orange peel in experimental dogs: A pilot study. Metabolites.

[9] Martignoni M., Groothuis G.M.M., de Kanter R. (2006). Species differences between mouse, rat, dog, monkey and human CYP-mediated drug metabolism, inhibition and induction. Expert Opin. Drug Metab. Toxicol..

[10] Bian Y., Sun M., Chen H., Ren G., Fu K., Yang N., Zhang M., Zhou N., Lu Y., Li N. (2022). Metabolites identification and species comparison of Oroxylin A, an anti-cancer flavonoid, in vitro and in vivo by HPLC-Q-TOF-MS/MS. Xenobiotica.

[11] Vitturini C., Cerquetella M., Spaterna A., Bazzano M., Marchegiani A. (2025). Diagnosis of canine cognitive dysfunction syndrome: A narrative review. Vet. Sci..

[12] Stockman J. (2024). Nutrition and aging in dogs and cats. Adv. Exp. Med. Biol..

[13] Cryan J.F., O’Riordan K.J., Sandhu K., Peterson V., Dinan T.G. (2020). The gut microbiome in neurological disorders. Lancet Neurol..

[14] Ghosh T.S., Shanahan F., O’Toole P.W. (2022). The gut microbiome as a modulator of healthy ageing. Nat. Rev. Gastroenterol. Hepatol..

[15] Ambrosini Y.M., Borcherding D., Kanthasamy A., Kim H.J., Willette A.A., Jergens A., Allenspach K., Mochel J.P. (2019). The gut-brain axis in neurodegenerative diseases and relevance of the canine model: A review. Front. Aging Neurosci..

[16] Ministry of Agriculture, Forestry and Fisheries, Japan Safety of Feeds and Pet Foods. https://www.maff.go.jp/e/policies/ap_health/petfood/index.html.

[17] Ministry of Education, Culture, Sports, Science and Technology Standard Tables of Food Composition in Japan–2020 (Eighth Revised Edition). https://www.mext.go.jp/a_menu/syokuhinseibun/mext_01110.html.

[18] National Institute of Biomedical Innovation, Health and Nutrition (NIBIOHN) Chenpi Assay Method: Hesperidin Quantification Using Methanol Reflux. http://mpdb.nibiohn.go.jp/mpdb-bin/view_jp_assay_data.cgi?id=42.

[19] Samota M.K., Kaur M., Sharma M., Sarita, Krishnan V., Thakur J., Rawat M., Phogat B., Guru P.N. (2023). Hesperidin from citrus peel waste: Extraction and its health implications. Qual. Assur. Saf. Crop. Foods.

[20] Wirianto M., Wang C.Y., Kim E., Koike N., Gomez-Gutierrez R., Nohara K., Escobedo G., Choi J.M., Han C., Yagita K. (2022). The clock modulator nobiletin mitigates astrogliosis-associated neuroinflammation and disease hallmarks in an Alzheimer’s disease model. FASEB J..

[21] Nagasawa M., Mitsui S., En S., Ohtani N., Ohta M., Sakuma Y., Onaka T., Mogi K., Kikusui T. (2015). Social evolution. Oxytocin-gaze positive loop and the coevolution of human-dog bonds. Science.

[22] Seki T. (2015). Clinical effects of the NChinpi on the cognitive impairment of patients with Alzheimer’s disease. Nihon Yakurigaku Zasshi.

[23] Nishino S., Fujiki Y., Sato T., Kato Y., Shirai R., Oizumi H., Yamamoto M., Ohbuchi K., Miyamoto Y., Mizoguchi K. (2022). Hesperetin, a citrus flavonoid, ameliorates inflammatory cytokine-mediated inhibition of oligodendroglial cell morphological differentiation. Neurol. Int..

[24] Ministry of Agriculture, Forestry and Fisheries, Japan Act on Ensuring of Safety of Pet Animals Feed. Act No. 83 of 2008, Last Version Act No. 68 of 2025. https://laws.e-gov.go.jp/law/420AC0000000083/.

[25] Consumer Affairs Agency, Japan Food Labeling Act. Act No. 70 of 2013, Last Version Act No. 97 of 2018. https://www.japaneselawtranslation.go.jp/en/laws/view/3649.

[26] Dwyer J.T., Coates P.M., Smith M.J. (2018). Dietary supplements: Regulatory challenges and research resources. Nutrients.

[27] Ministry of Agriculture, Forestry and Fisheries, Japan Summary of Results for Citrus Production. https://www.maff.go.jp/j/tokei/kouhyou/sakumotu/sakkyou_kazyu/index.html.

[28] e-Stat Crop Statistics Survey. https://www.e-stat.go.jp/dbview?sid=0003313868.

[29] Bertero A., Fossati P., Caloni F. (2020). Indoor companion animal poisoning by plants in Europe. Front. Vet. Sci..

[30] Ministry of Health, Labour and Welfare, Japan Act on Securing Quality, Efficacy and Safety of Products Including Pharmaceuticals and Medical Devices. Act No. 145 of 1960, Last Version Act No. 50 of 2015. https://www.japaneselawtranslation.go.jp/en/laws/view/3213.

[31] Mandour D.A., Bendary M.A., Alsemeh A.E. (2021). Histological and imunohistochemical alterations of hippocampus and prefrontal cortex in a rat model of Alzheimer like-disease with a preferential role of the flavonoid “hesperidin”. J. Mol. Histol..

[32] Tamilselvam K., Braidy N., Manivasagam T., Essa M.M., Prasad N.R., Karthikeyan S., Guillemin G.J. (2013). Neuroprotective effects of hesperidin, a plant flavanone, on rotenone-induced oxidative stress and apoptosis in a cellular model for Parkinson’s disease. Oxid. Med. Cell Longev..

[33] Kumar P., Kumar A. (2010). Protective effect of hesperidin and naringin against 3-nitropropionic acid induced Huntington’s like symptoms in rats: Possible role of nitric oxide. Behav. Brain. Res..

[34] Ikemura M., Sasaki Y., Giddings J.C., Yamamoto J. (2012). Preventive effects of hesperidin, glucosyl hesperidin and naringin on hypertension and cerebral thrombosis in stroke-prone spontaneously hypertensive rats. Phytother. Res..

[35] Raza S.S., Khan M.M., Ahmad A., Ashafaq M., Khuwaja G., Tabassum R., Islam F. (2011). Hesperidin ameliorates functional and histological outcome and reduces neuroinflammation in experimental stroke. Brain Res..

[36] Salem H.R.A., El-Raouf A.A., Saleh E.M., Shalaby K.A. (2012). Influence of hesperidin combined with Sinemet on genetical and biochemical abnormalities in rats suffering from Parkinson’s disease. Life Sci. J..

[37] Matsumoto H., Ikoma Y., Sugiura M., Yano M., Hasegawa Y. (2004). Identification and quantification of the conjugated metabolites derived from orally administered hesperidin in rat plasma. J. Agric. Food Chem..

