# Supplementary material for: Preliminary Safety Assessment for Mandarin Orange Peel Administration to Dogs Based on Physical Conditions and Blood Examination Parameters
Source: Metabolites. 2026 Mar 23;16(3):213. doi: 10.3390/metabo16030213 (PMC13027855; doi:10.3390/metabo16030213)
Supplement: Supplementary file 1 [file metabolites-16-00213-s001.zip › metabolites-4182070--Supplementary.pdf]

Table S1. List of Compounds for Residual Pesticide Analysis (202 Compounds)

Acrinathrin	Metalaxyl and Mefenoxam	Chlorfuazuron	Fludioxonil	Pyflubumide
Benfuracarb	Methidathion	Chloridazon	Flufenacet	Pyraclostrobin
Bifenazate	Oxpoconazole Fumarate	Chromafenozide	Flufenoxuron	Pyrafufen-ethyl
Bifenthrin	Paclobutrazol	Clofentezine	Flumioxazin	Pyrazifumid
Bitertanol	Pendimethalin	Clothianidin	Fluopicolide	Pyrazolynate
Bromacil	Permethrin	Cyanazine	Fluxametamide	Pyrazoxyfen
Butachlor	Phenthoate	Cyantraniliprole	Halosulfuron-methyl	Pyribencarb
Cadusafos	Pirimiphos-methyl	Cyazofamid	Hexaconazole	Pyrifuquinazon
Carbosulfan	Procymidone	Cyclaniliprole	Hexythiazox (any ratio of constituent isomers)	Pyriminobac-methyl
Chlorfenapyr	Prohydrojasmone	Cyenopyrafen	Imazamox-Ammonium	Pyroxasulfone
Chlorpropham	Propiconazole	Cyfufenamid	Imibenconazole	Quizalofop-ethyl
Chlorpyrifos	Prothiofos	Cyfumetofen	Imidacloprid	Sethoxydim
Cyanophos	Pyridaben	Cymoxanil	Indoxacarb	Simazine
Cyfuthrin	Pyridalyl	Cyprodinil	Inpyrfuxam	Simeconazole
Cyhalothrin	Pyrimidifen	Daimuron	Ipconazole	Spinetoram
Cypermethrin	Pyriproxyfen	Dichlorprop	Ipfufenoquin	Spinosad
Cyproconazole	Silafuofen	Diclocymet	Isofetamid	Spiromesifen
Deltamethrin/Tralomethrin	Spirodiclofen	Difubenzuron	Isoprothiolane	Spirotetramat
Diazinon	Tebufenpyrad	Dimethenamid	Isouron	Sulfoxafor
Dichlobenil	Tebuconazole	Dimethomorph (sum of isomers)	Kresoxim-methyl	Tebufenozide
Diethofencarb	Tefuthrin	Dinotefuran	Lufenuron	Tefubenzuron
Difenoconazole	Terbacil	Diuron	Mandestrobin	Tefuryltrione
Difufenican	Tetradifon	Emamectin benzoate	Mandipropamid	Tepraloxydim
Dimethoate	Thiobencarb	Ethiprole	MCPB	Tetraconazole
Disulfoton	Tolclofos-methyl	Etoxazole	Mepanipyrim	Thiacloprid
Etofenprox	Trifumizole	Ethychlozate	Metafumizone	Thiamethoxam
Fenarimol	Trifuralin	Famoxadone	Metconazole	Thifensulfuron-methyl
Fenitrothion	2,4-Dichlorophenoxyacetic acid	Fenamidone	Methoxyfenozide	Thifuzamide
Fenobucarb	6-Benzyladenine	Fenbuconazole	Milbemectin (sum)	Thiodicarb and Methomyl
Fenpropathrin	Abamectin (Sum)	Fenhexamid	Napropamide	Tiadinil
Fenthion	Acetamiprid	Fenothiocarb	Nicosulfuron	Tolfenpyrad
Fenvalerate	Alanycarb	Fenoxanil	Nitenpyram	Trichlorfon
Fipronil	Amisulbrom	Fenpyrazamine	Novaluron	Triclopyr
Fluopyram	Azoxystrobin	Flazasulfuron	Oxadiazon	Tricyclazole
Fluvalinate	Benthiavalicarb, isopropyl-	Flometoquin	Penthiopyrad	Trifloxystrobin
Fluxapyroxad	Boscalid	Fenpyroximate	Picarbutrazox	Trinexapac-ethyl
Ipfencarbazone	Buprofezin	Flonicamid	Picoxystrobin	Dithianon
Iprobenfos	Carbaryl	Fluacrypyrim	Prochloraz	Dithiocarbamates*
Iprodione	Carbendazim/Benomyl/ Thiophanate-methyl (total)	Fluazifop-butyl	Profenofos	
Isoxathion	Carfentrazone-ethyl	Fluazinam	Propargite	
Malathion	Chlorantraniliprole	Flubendiamide	Pymetrozine	

* dithiocarbamates expressed as CS2, including maneb, mancozeb, metiram, propineb, thiram and ziram

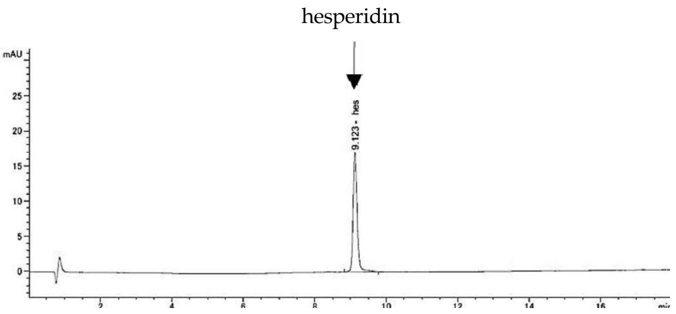

Standard Solution

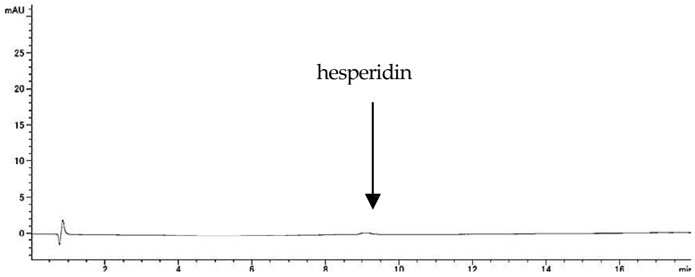

Blank Solution

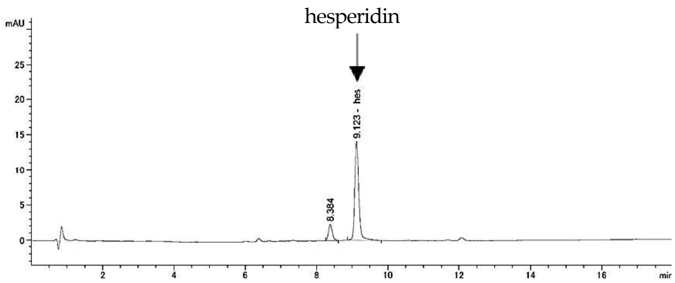

Sample Solution

Figure S1. HPLC Chromatogram of Hesperidin

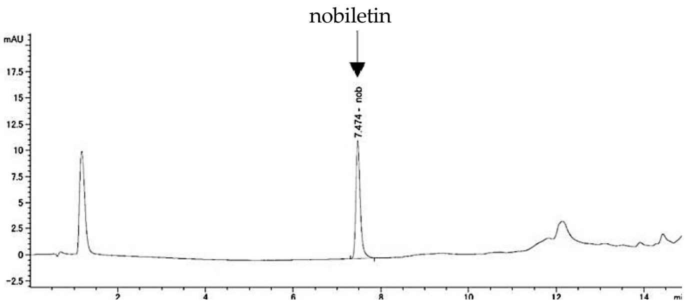

Standard Solution

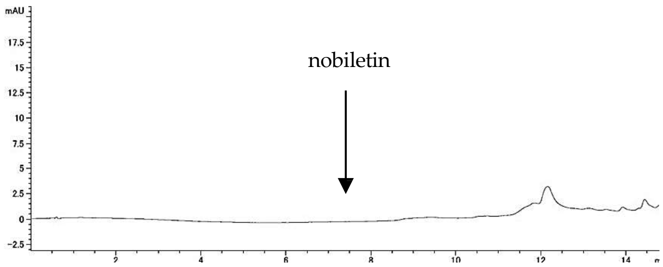

Blank Solution

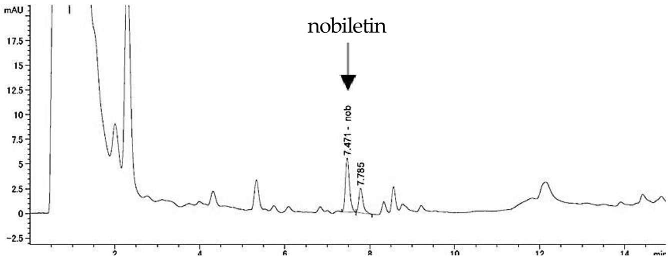

Sample Solution

Figure S2. HPLC Chromatogram of Nobiletin

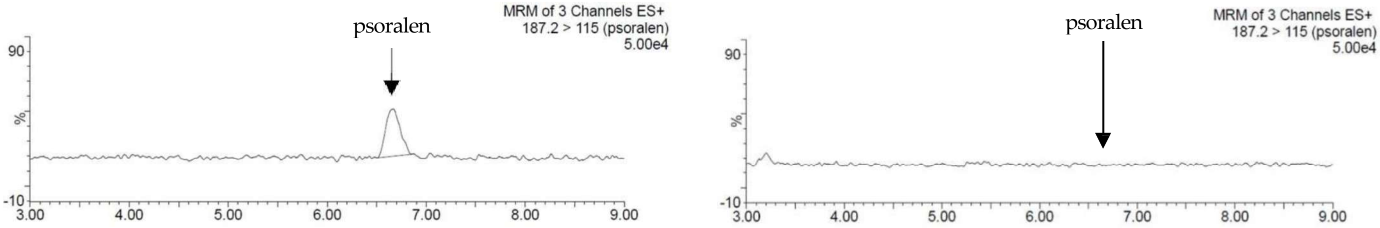

Standard Solution

Sample Solution

Figure S3. LC/MS Chromatogram of Psoralen

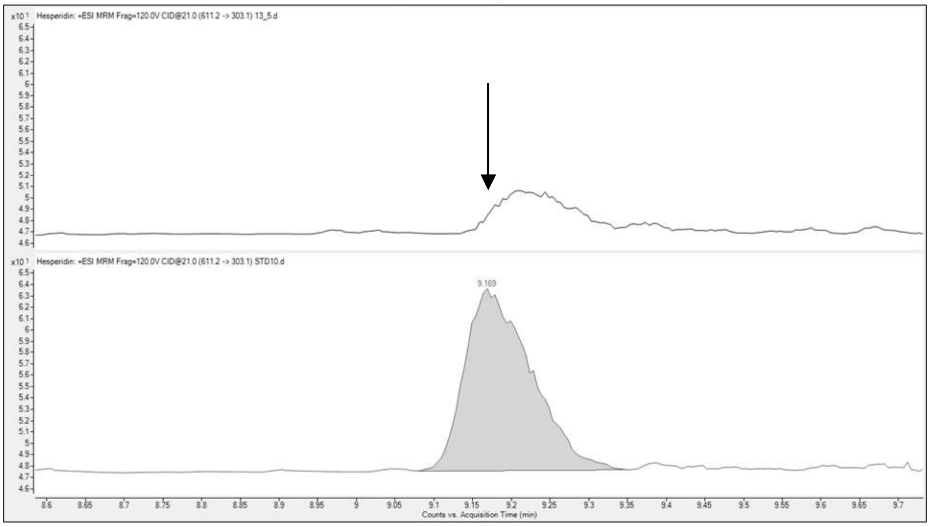

Typical chromatograms of the sample solution (upper) and the hesperidin standard solution (lower)

(m/z 611.2 → 303.1)

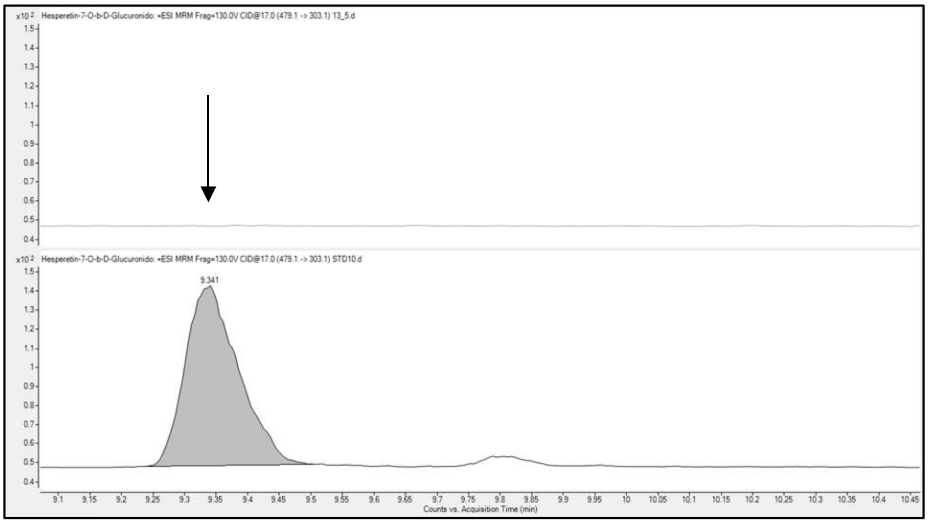

Typical chromatograms of the sample solution (upper)

and the rac-hesperetin 7-O-β-D-glucuronide standard solution (lower)

(m/z 479.1 → 303.1)

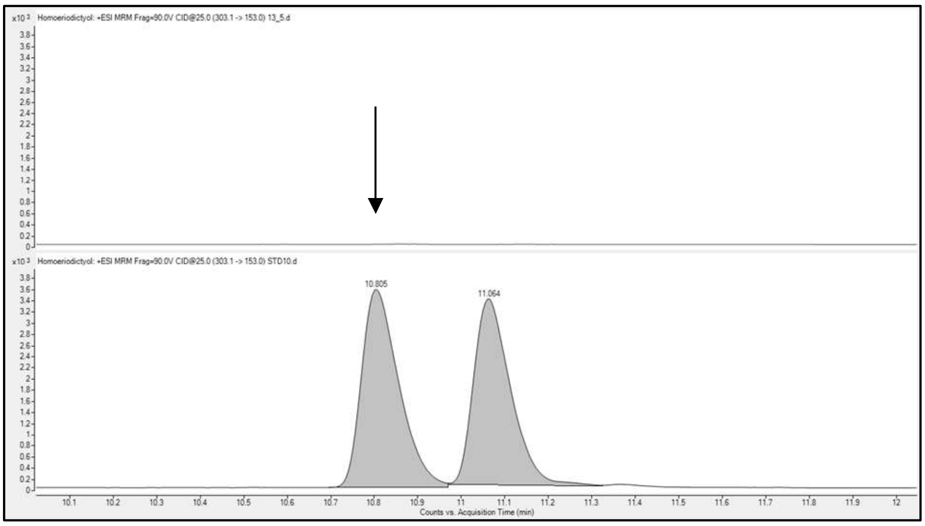

Typical chromatograms of the sample solution (upper)and the homoeriodictyol standard solution (lower)
(m/z 313.1 → 153.0)

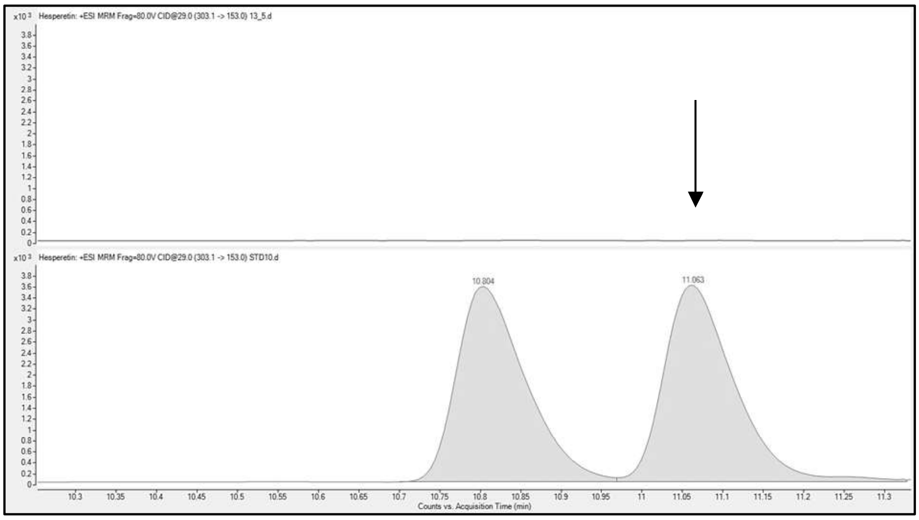

Typical chromatograms of the sample solution (upper) and the hesperetin standard solution (lower)
(m/z 303.1 → 153.0)

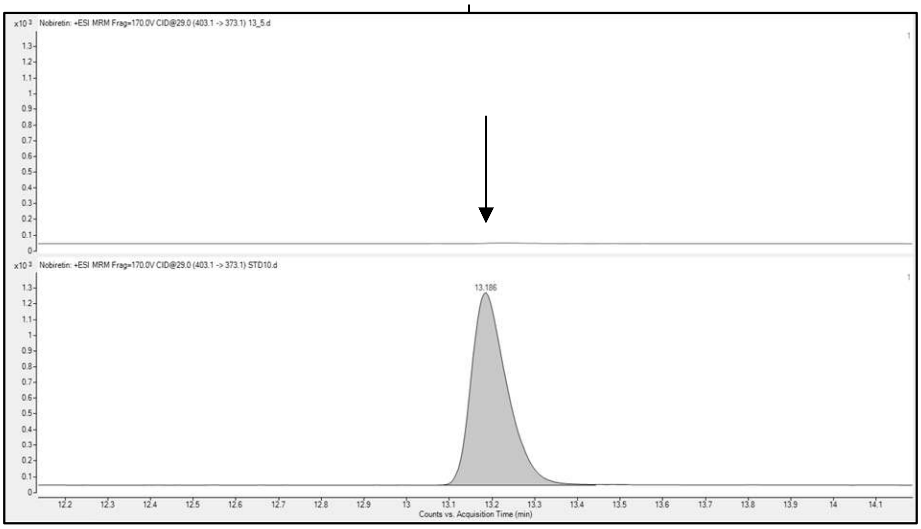

Typical chromatograms of the sample solution (upper) and the nobiletin standard solution (lower)
(m/z 403.1 → 373.1)

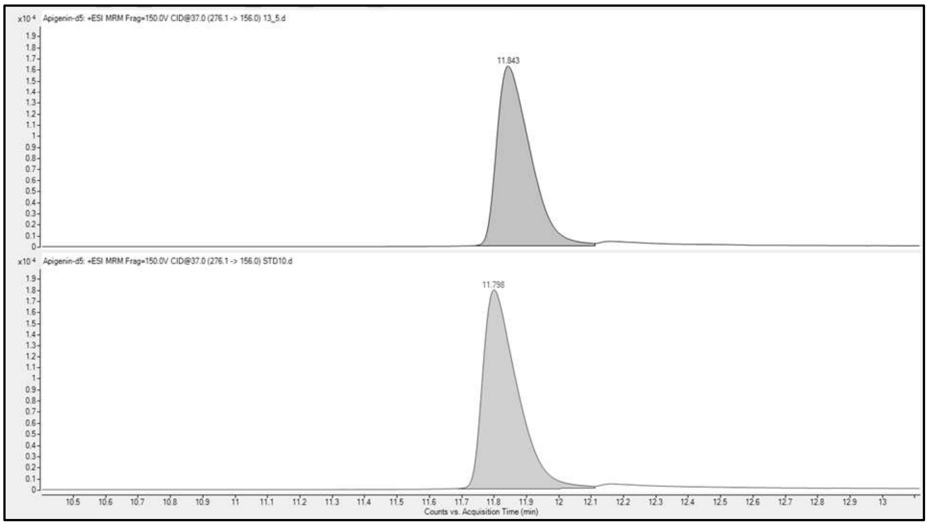

Typical chromatograms of the sample solution (upper) and the standard solution (lower), both containing apigenin-d5 as an internal standard (m/z 276.1 → 156.0)

Figure S4. LC/MS Chromatogram from CSF Analysis
